# Exploring Risk Factors and Neurophysiological Mechanisms Underlying the Development of Chronic Postsurgical Pain After Thoracic Surgery: Protocol for an Observational Feasibility Study

**DOI:** 10.2196/81042

**Published:** 2026-01-12

**Authors:** Mustaali Hussain, Stevie Dante Foglia, Ameer Hamoodi, Jinhui Ma, John Agzarian, Christian John Finley, Yaron Shargall, Aimee Jennifer Nelson, Harsha Shanthanna

**Affiliations:** 1 Department of Kinesiology Faculty of Science McMaster University Hamilton, ON Canada; 2 School of Biomedical Engineering McMaster University Hamilton, ON Canada; 3 Department of Health Research Methods, Evidence, and Impact Faculty of Health Sciences McMaster University Hamilton, ON Canada; 4 Division of Thoracic Surgery Faculty of Health Sciences McMaster University Hamilton, ON Canada; 5 Department of Anesthesia Faculty of Health Sciences McMaster University Hamilton, ON Canada

**Keywords:** chronic postsurgical pain, thoracic surgery, transcranial magnetic stimulation, TMS, electroencephalography, EEG, risk factors, neurophysiological mechanisms

## Abstract

**Background:**

Chronic postsurgical pain (CPSP) is a debilitating chronic pain condition that particularly impacts patients undergoing thoracic surgery, with incidence rates of up to 50%. The current understanding of risk factors is limited, and preoperative neurophysiological risk factors that may predict the development of CPSP have not yet been explored. Additionally, the specific neural mechanisms underlying the transition to CPSP are not well characterized. As a novel approach, we propose the use of transcranial magnetic stimulation and electroencephalography, along with other patient and surgical factors, to understand the neurophysiological mechanisms underlying the onset of CPSP after thoracic surgery.

**Objective:**

The primary objective of this study is to evaluate the feasibility of our study design to inform a larger observational cohort study. Secondary objectives include exploring preoperative neurophysiological markers along with clinical characteristics associated with a higher risk of developing CPSP, as well as exploring postoperative differences in cortical function between patients who undergo thoracic surgery and develop CPSP compared with those who do not develop CPSP.

**Methods:**

A total of 30 participants undergoing video-assisted thoracic surgery or a robotic-assisted thoracic lobectomy, wedge resection, segmental section, or minimally invasive esophagectomy, will be recruited to take part in 2 assessment sessions. The first assessment will take place 2 to 3 weeks before surgery, and the second assessment will take place 3 months after surgery, during which the CPSP diagnosis of each participant will be assessed by the experimenter using a validated definition. Feasibility outcomes include recruitment and retention rates of study participants. The secondary objectives include exploring factors associated with the development of CPSP, as well as examining postoperative differences in neurophysiological measures between patients with and without CPSP. We will consider the following neurophysiological measures for these objectives: transcranial magnetic stimulation measures of short-latency intracortical inhibition, cortical silent period, and motor evoked potentials; electroencephalography measures of resting band activity, event-related desynchronization, and corticomuscular coherence; and quantitative sensory testing of mechanical detection threshold and pressure pain threshold.

**Results:**

This is an ethics-approved, ongoing study. Initial funding for this study was provided in March 2023. Recruitment for the study began in January 2025. A total of 22 participants have been recruited for the study. We anticipate completing data collection for this study by April 2026, with data analysis to follow.

**Conclusions:**

This protocol details our study design for a feasibility study exploring the neurophysiological markers and patient characteristics associated with the development of CPSP. Demonstration of feasibility is expected to lead to a larger study. Improved understanding of the risk factors and mechanisms underlying CPSP may inform the delivery of targeted therapies and preventive measures to reduce the incidence of CPSP after thoracic surgery.

**International Registered Report Identifier (IRRID):**

DERR1-10.2196/81042

## Introduction

Chronic postsurgical pain (CPSP) is defined by the International Association for the Study of Pain as pain localized to the operated area that persists for at least 3 months after surgery and cannot be explained by other potential causes [1]. CPSP is a debilitating condition that negatively impacts patients’ quality of life [2,3], while also placing a significant burden on health care providers and hospitals [2]. While the incidence of CPSP can vary across surgical procedures, thoracic surgery shows a markedly high rate, with rates between 30% and 50% [4-7].

Understanding the preoperative profile that predicts the development of CPSP is important to identify patients at higher risk and implement early interventions. Exploring neurophysiological risk factors using transcranial magnetic stimulation (TMS) and electroencephalography (EEG) may improve our understanding beyond the currently identified risk factors, such as female sex [6,8], depression and anxiety [5,8], younger age, and preoperative pain [8-10], which alone show only moderate prognostic value [11]. In previous work, measures of cortical function via TMS or EEG have successfully predicted the development of chronic neuropathic pain [12] and other neurological conditions [13].

These neurophysiological tools may also provide insight into the maladaptive mechanisms underlying the persistence of CPSP after thoracic surgery. While central sensitization of pain processing centers to nociceptive input has been the prevailing theory behind the persistence of CPSP [14], the specific dysregulations involved remain incompletely understood. In other chronic pain conditions, TMS assessments have revealed reductions in cortical excitability and intracortical inhibition [15-19], suggesting impairments in glutamatergic and γ-aminobutyric acid-mediated neural transmission. These markers may also explain the associations between depression and the risk of CPSP [20] but are unlikely to account for other risk factors, such as younger age [21]. EEG assessments among individuals with various chronic pain conditions have demonstrated elevated band power across various frequency bands at rest [22-27] and elevated cortical desynchronization during movements across various cortical regions [25,28], which indicate impairments in sensory processing. Such EEG markers may also provide insight into maladaptive cognitive states, such as pain catastrophizing, another risk factor for CPSP [8]. Collectively, these findings indicate that TMS and EEG measurements may provide valuable insights into the neurophysiological risk factors and mechanisms underlying the development and presence of CPSP after thoracic surgery.

Beyond these mechanistic aims, an important consideration is assessing the feasibility of longitudinally studying patients undergoing thoracic surgery before and after surgery. Given that these individuals have been shown to exhibit higher levels of fear of surgery preoperatively compared with those undergoing other surgeries [29], the ability to recruit them for a neurophysiological assessment study may present challenges, due to their elevated concerns regarding the surgery itself. In addition, the fact that thoracic surgery leads to postoperative complications in up to one-third of patients [30] alongside hospital readmission rates of 10% to 20% up to 90 days after surgery [30] suggests that performing follow-up neurophysiological testing on these patients after surgery may also be challenging and lead to high attrition rates.

Therefore, the primary objective of this study is to assess the feasibility of recruitment and retention of patients undergoing thoracic surgery before and after surgery, which will help inform the planning of a larger cohort study. Secondary objectives for this pilot study include exploring preoperative neurophysiological markers (in conjunction with clinical profile characteristics) associated with a higher risk of CPSP, as well as identifying postoperative differences in cortical function between patients who develop CPSP (CPSP+) and those who do not develop CPSP (CPSP–).

## Methods

### Recruitment

This study will recruit 30 participants aged between 18 and 80 years who are scheduled to undergo video-assisted thoracoscopic surgery or a robotic-assisted thoracic lobectomy, wedge resection, segmental resection, or a minimally invasive esophagectomy (MIE) at St Joseph’s Healthcare (SJH; Hamilton, Ontario). Although open thoracotomies and esophagectomies are associated with a higher risk of CPSP, they are not commonly performed. Participants must have comprehension of the English language or have an interpreter present to participate. Participants will be excluded if (1) they have any contraindications to undergoing TMS, including (but not limited to) the presence of a pacemaker, metal, electrical, or magnetic implants near the head or neck (not including titanium); a known history of untreated or uncontrolled psychological disorders, pregnancy, history of seizure, or a diagnosis of epilepsy, or if they are taking prescription medications that increase the risk of seizure; (2) they have a known significant psychological impairment that may affect comprehension of the study’s instructions or their ability to participate; or (3) they are participating in another research study intervention that may impact the development of CPSP. The presence of other comorbidities or pre-existing pain conditions will not be a reason for exclusion from our study.

The research team will obtain a rolling list of patients consented for surgery from the thoracic surgery team at SJH, and these patients will subsequently be contacted to be given a detailed explanation of the study protocol and to assess eligibility through completing the TMS screening questionnaire. If eligible and willing to participate, participants will be invited to complete a written informed consent form. Participants will be informed that they can withdraw at any time during the study period. Further, they have the right to withdraw their data from the study for up to 2 weeks following their withdrawal or completion of the study. All assessment sessions will be conducted at the McMaster University.

### Ethical Considerations

This study will be conducted in compliance with the protocol and the principles laid down in the Declaration of Helsinki. Approval to conduct this study has been granted by the Hamilton Integrated Research Ethics Board (17806). Participants will provide written informed consent prior to participation, and they will be made aware that they may withdraw at any time during the study period.

### Study Design

**Figure 1:**
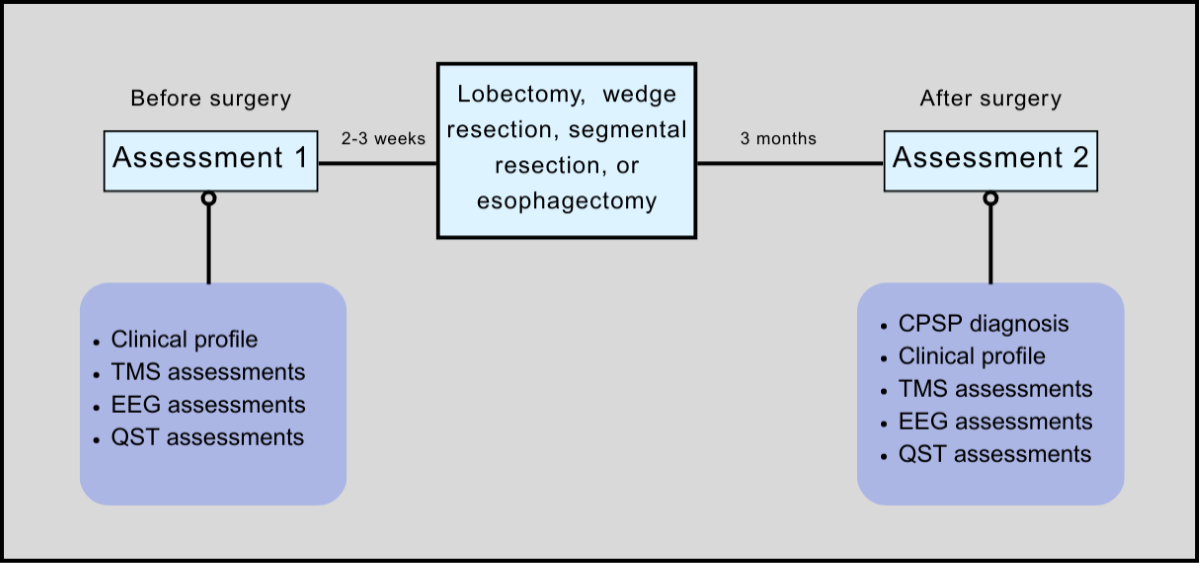
Experimental timeline of the study. All participants will take part in 2 assessments. Assessment 1 (T0) will occur 2-3 weeks before the participants’ scheduled procedure. Participants will then complete assessment 2 (T1) at 3 months after surgery. Both assessments will involve collecting clinical profile information using questionnaires, neurophysiological measures acquired using transcranial magnetic stimulation (TMS) and electroencephalography (EEG), and measures of sensation with quantitative sensory testing (QST). See table 1 for the specific measurements acquired. Diagnosis of chronic postsurgical pain (CPSP) will be evaluated in the second assessment. Each assessment will take approximately 3 hours to complete.

This is an observational feasibility study in individuals undergoing video-assisted thoracic surgery or robotic-assisted thoracic lobectomy, wedge resection, segmental resection, or MIE. Participants will attend 2 assessment sessions: the first will occur 2 to 3 weeks before their scheduled procedure, and the second will take place 3 months after surgery (Figure 1). Each session will span about 3 hours and will be scheduled flexibly, with ample breaks provided as needed to minimize participant burden.

### Study Outcomes

#### Feasibility

The primary objective is to assess the feasibility of the study design. Feasibility outcomes will include (1) participant recruitment and (2) participant retention for the postsurgical assessment. On the basis of past records of the thoracic surgery division at SJH, we aim to recruit 3 to 4 patients per month over a 10-month study period. We will consider monthly recruitment of 3 to 4 patients and >90% retention to be feasible, monthly recruitment of 1 to 2 patients and 85% to 90% retention as feasible with appropriate modifications, and no patients recruited in 5 out of 10 months and <85% retention as not feasible. These cutoffs will be used to evaluate the need for protocol revisions before conducting a large-scale study.

#### Demographic and Clinical Information

In the presurgical assessment, information regarding each participant’s age, sex, history of prior surgery, history of preexisting pain, reason for upcoming surgery, other comorbidities, adjuvant therapies, and pain medication use will be documented. In the postsurgical assessment, details of each patient’s surgery type, number of ports, adjuvant therapies, and use of pain medications will be documented by the experimenter. In addition, the anesthesia protocols used for each participant’s surgery will be collected through electronic chart data, including the anesthetic technique (volatile or total intravenous), regional anesthesia type (thoracic epidural, paravertebral, erector spinae plane, or intercostal block), delivery mode (single-shot or continuous catheter), and duration of postoperative infusion.

This information will be reported descriptively and considered during data analysis so that its potential impact can be considered in the interpretation of the results.

#### Questionnaires

The questionnaires that will be completed in a self-administered manner during each assessment session are presented in Textbox 1.

**Questionnaires.**

**Patient-Reported Outcomes Measurement Information System-29 (PROMIS-29; v2.0)**

The PROMIS-29 questionnaire will be used to assess 7 health domains, including physical function, anxiety, depression, fatigue, sleep disturbances, ability to participate in social roles and activities, and pain interference [31]. The 0 to 10 numerical rating scale included in the PROMIS will be used to assess the presence and severity of pain. Scores from this questionnaire will be expressed as *t* scores, which are standardized relative to a reference healthy population (mean 50, SD 10). Higher scores correspond to increased levels of the measured domain [32]. This questionnaire was chosen as it can efficiently capture a broad range of health domains.

**Patient Health Questionnaire-4 (PHQ-4)**

The PHQ-4 [33] is a 4-item self-report screening questionnaire for depression and anxiety symptoms experienced in the past 2 weeks, consisting of the Generalized Anxiety Disorder-2 (GAD-2; 2 core criteria for generalized anxiety disorder) [34] and the PHQ-2 (2 core criteria for depression) [35]. This will be used to assess symptoms of depression and anxiety. A score of ≥3 for GAD-2 suggests the presence of anxiety, and a score of ≥3 for PHQ-2 suggests the presence of depression. Given that depression and anxiety are cited as potential risk factors for developing chronic postsurgical pain [5,8], depression and anxiety levels assessed from these questionnaires will also be incorporated as features within our analysis. This questionnaire was selected for its validity [36] and its ultrabrief design to minimize participant burden.

#### TMS Assessments

#### Overview

TMS will be performed using a 50-mm figure-of-8 coil attached to a Magstim 200^2^ stimulator (Magstim). This study will focus on stimulating the motor hot spot of the right first dorsal interosseus (FDI) muscle for all TMS measures. Surface electrodes (9 mm Ag-AgCl) will be used to record electromyography (EMG) activity from the FDI muscle of the right hand.

Resting motor threshold, defined as the lowest intensity required to evoke a motor evoked potential (MEP) ≥50 μV in 50% of the time, will be determined for each participant’s FDI muscle [37]. Active motor threshold, defined as the lowest intensity required to evoke an MEP≥200-μV in 5 out of 10 consecutive trials [37] while participants maintain a sustained isometric contraction of the right FDI muscle to 10% of their maximum voluntary contraction (MVC), will also be recorded. This MVC value will be determined beforehand, via participants performing a brief 5-second isometric voluntary maximum contraction of the right FDI muscle, with maximal EMG activity recorded.

#### MEP Analysis

MEPs will be acquired by stimulating the motor hot spot for the right FDI muscle at 120% of the resting motor threshold for a total of 30 pulses at rest. MEPs will also be collected during voluntary contraction of the target FDI muscle to 50% MVC, with 20 pulses delivered at an intensity that evokes a resting MEP with approximately 1 mV peak-to-peak amplitude (determined beforehand). Average MEP amplitude will be calculated for each condition, and this measure will be used to assess the levels of corticospinal excitability.

#### Short-Latency Intracortical Inhibition

Short-latency intracortical inhibition (SICI), a measure of cortical inhibition, is measured using paired-pulse TMS with an interstimulus interval of 2 ms between the conditioning stimulus (CS) and test stimulus (TS). The CS will be set to 90% of the active motor threshold, and the TS will be set to the intensity evoking an MEP with a peak-peak amplitude of approximately 1 mV in the right FDI. SICI will be calculated as the ratio of the average conditioned MEP (CS-TS) to the amplitude of the average unconditioned MEP (MEP-TS only), with smaller values indicating greater levels of intracortical inhibition, and vice versa. A total of 25 TS and 25 CS+TS pulses will be delivered.

#### Cortical Silent Period

Cortical silent period (CSP), a measure of spinal and cortical inhibition, is an interruption in the ongoing voluntary EMG signal that occurs following an MEP evoked by a suprathreshold TMS pulse. The CSP will be probed by delivering single-pulse TMS during voluntary contraction at approximately 50% MVC of the FDI. A total of 20 pulses will be delivered at an intensity evoking a resting MEP with a peak-peak amplitude of approximately 1 mV (determined beforehand). CSP duration will be measured for each trial and averaged. CSP onset will be defined as the beginning of the TMS-evoked MEP, and CSP offset will be defined as when the voluntary EMG signal reappears. Once CSP onset and offset are determined, CSP duration will be calculated.

#### Quantitative Sensory Testing

Quantitative sensory testing (QST) will be used in this study to assess somatosensory function before and after surgery. The series of tests to be used includes the mechanical detection threshold (MDT) and the pressure pain threshold (PPT) tests. For patients undergoing lobectomy, wedge resection, or segmental resection, QST will be performed unilaterally at 3 sites on the sides of the trunk (approximately the fourth, seventh, and ninth intercostal spaces) [38,39]. For patients undergoing MIE, QST will be performed bilaterally at 3 sites on the sides of the trunk (approximately the fourth, seventh, and ninth intercostal spaces) and 1 site at the abdomen (around the navel) [40]. QST results will be analyzed in their original measurement units (lb for PPT and mN for MDT).

#### EEG Assessments

EEG will be recorded using an active EEG electrode system (Quik-Cap Neo Net; Compumedics Neuroscan) connected to a Grael 4K EEG amplifier (Compumedics Neuroscan) with 32 channels of recording electrodes positioned across the cortical hemisphere using the international 10 to 20 system. EEG data will be used to derive the following 3 outcomes.

#### Band Activity

Spontaneous EEG will be recorded under 2 conditions: eyes open (EO) and eyes closed (EC). Each condition will last 5 minutes. During the EO condition, a white fixation cross will be presented at the center of the screen for patients to focus on. During the EC condition, patients will sit relaxed with their eyes closed. Experimenters will ensure that patients remain awake during the EC condition through continuous monitoring.

Following findings from Vuckovic et al [41], features will be based on the power spectral density calculated for each frequency band (alpha [8-12 Hz] and beta [13-30 Hz]) for the EO and EC tasks. Additionally, the ratio between the EC and EO states for each frequency band will be calculated [41]. Features will be computed for each of the 32 channels.

#### Event-Related Desynchronization

Event-related desynchronization (ERD) will be measured during periodic submaximal isometric contractions of the abductor pollicis brevis muscle.

ERD measures the reduction in the power of synchronized neural oscillations across various frequency bands in response to movement and serves as a marker of increased cortical excitability [28]. ERD will be assessed using an EEG recorded from electrodes positioned over the primary motor cortex (C3 and C4). ERD will be calculated by comparing alpha and beta band power during the active movement phase (0.5-1.5 seconds) to a premovement baseline period (−3.5 to −0.5 s), using standard event-related spectral perturbation analysis [42].

#### Corticomuscular Coherence

Corticomuscular coherence (CMC) will be measured during periodic submaximal isometric contractions of the abductor pollicis brevis muscle.

CMC is a measure of the synchrony between oscillations in EEG signals and EMG signals at the muscle, suggested to reflect reciprocal communication between the motor cortex and active muscle during movement [43]. Deficits in CMC have been shown to reflect impairments in sensorimotor integration in chronic pain [44]. In this study, CMC will be calculated from EEG recordings over C3 and C4 and EMG signals from the right abductor pollicis brevis muscle using established correlation-based methods [45], focusing on the mu (8-12 Hz) and beta (15-30 Hz) frequency bands.

#### Measurements Acquired in Each Assessment

The measurements acquired during each assessment are summarized in Table 1. Each of these TMS and EEG measures used has shown good to excellent within-session or between-session reliability [46-51], and therefore provides a reliable basis for evaluating cortical activity, excitability, and inhibition.

**Table 1:** Measurements acquired in each assessment.

Assessment	Outcomes measured
T0 (before surgery)	Demographic and clinical information (age, sex, surgery history, comorbidities, adjuvant therapies, and pain medications) Questionnaires (PROMIS-29^a^ v2.1 and PHQ-4^b^) TMS^c^ (MEPs^d^, SICI^e^, and CSP^f^) EEG^g^ (band activity, ERD^h^, and CMC^i^) QST^j^ (MDT^k^ and PPT^l^)
T1 (3 months after surgery)	Demographic and clinical information (age, sex, surgery history, surgery type, number of ports, comorbidities, adjuvant therapies, and pain medications) Questionnaires (PROMIS-29 v2.1 and PHQ-4) TMS (MEPs, SICI, and CSP) EEG (band activity, ERD, and CMC) QST (MDT and PPT)

^a^PROMIS-29: Patient-Reported Outcomes Measurement Information System–29.

^b^PHQ-4: Patient Health Questionnaire-4.

^c^TMS: transcranial magnetic stimulation.

^d^MEP: motor evoked potential.

^e^SICI: short-latency intracortical inhibition.

^f^CSP: cortical silent period.

^g^EEG: electroencephalography.

^h^ERD: event-related desynchronization.

^i^CMC: corticomuscular coherence.

^j^QST: quantitative sensory testing.

^k^MDT: mechanical detection threshold.

^l^PPT: pressure pain threshold.

#### Diagnosis of CPSP

At T1, participants will be categorized as CPSP+ or CPSP–. CPSP– will be defined as a participant experiencing no persistent pain related to the surgery 3 months after the operation. CPSP+ will be defined as the presence of new persistent pain that is localized to the operated area (of any severity) or pain of increased severity (if there was pre-existing pain in that site) 3 months after the operation, which cannot be attributable to any other identifiable cause. This diagnosis will be evaluated by asking the following question: Do you have persistent pain (that started or increased with or after surgery) at or near the surgical area? For patients who answer yes, the severity of pain localized to the surgical area will be assessed using the numeric rating scale [52].

### Statistical Analysis

#### Power

The sample size for this study is based on evaluating feasibility for a larger study, and thus is not sufficiently powered to detect clinical differences. We based our sample size on pilot trial sample size calculations using a CI approach suggested by Thabane et al [53]. In addition, our sample size was determined in consultation with the thoracic surgery team at SJH, who provided data on the number of surgeries performed within the department.

#### Identifying Prospective Markers Associated With CPSP Development

Univariable logistic regression will be conducted to explore the associations between preoperative and perioperative characteristics and CPSP status. These analyses will be considered hypothesis-generating to inform the planned larger cohort study. We will test the individual association of the following independent variables with the diagnosis of CPSP: demographic and clinical information (age, sex, baseline pain intensity, depression and anxiety levels, surgery type, and regional anesthesia information), TMS measures (SICI, CSP, and MEPs), EEG measures (band activity, ERD, and CMC), and QST measures (MDT and PPT). Variables that show a statistically significant relationship (*P*<.05) will be considered independent predictors of CPSP development in this pilot study. Odds ratios with 95% CIs will also be reported.

#### Identifying Neurophysiological Mechanisms Underlying the Presence of CPSP

Exploratory comparisons of postsurgical TMS and EEG measures between patients who are CPSP+ and CPSP– will be conducted using 2-sample 2-tailed *t* tests. Significance will be set to α=.05, and effect sizes (Cohen *d*) will be reported with 95% CIs. Before this analysis, normality will be assessed with the Shapiro-Wilk test, and outliers will be removed with Grubbs test.

These secondary exploratory aims will be analyzed using an available-case approach, whereby participants with missing data for a given variable will be excluded only from analyses involving that variable and retained in all others.

## Results

Initial funding for this study was provided in March 2023. Recruitment for the study began in January 2025. A total of 22 participants have been enrolled in the study and have completed the presurgical assessment. Of these, 10 participants have completed the postsurgical assessment. We anticipate completing data collection for this study by April 2026, with data analysis to follow.

## Discussion

Understanding and mitigating the high incidence of CPSP after thoracic surgery remains an unmet medical need. This pilot study takes a novel approach by investigating the potential neurophysiological risk factors and mechanisms underlying its development. This pilot study will allow for an understanding of the feasibility of conducting such an investigation on a large scale, through assessing participant recruitment and retention rates, which may allow us to identify potential recruitment or study design challenges that can lead to attrition and burden. Prior use of TMS [15,18,19,54-57] and EEG [22-25] to successfully identify impairments in cortical function in other chronic pain conditions supports the potential of our work to improve the understanding of factors that increase the risk of developing CPSP after thoracic surgery, as well as to advance knowledge on neural mechanisms underlying its presence. Such an investigation may serve to support the understanding of CPSP to improve preoperative identification and prevention, as well as inform the development of effective treatment strategies.

This study has several limitations. As a single-center feasibility study with a sample size of 30 participants, the analyses will not be powered for any definitive conclusions. In addition, pain trajectories during the immediate postoperative period and longer-term follow-up beyond 3 months will not be assessed due to logistical constraints. In addition, the assessment of CPSP status did not include further information on pain categorization. These are important considerations for future studies to strengthen their clinical relevance. The neurophysiological assessments used here are time-intensive and largely confined to research settings. However, they are essential for establishing a mechanistic understanding, which may inform more simplified, bedside-compatible assessments in the future, such as bedside EEG. Despite these limitations, this study will provide critical feasibility data and preliminary mechanistic insight to guide the design of a larger cohort study examining the neurophysiological mechanisms of CPSP.

## Abbreviations

CMC: corticomuscular coherence
CPSP: chronic postsurgical pain
CS: conditioning stimulus
CSP: cortical silent period
EC: eyes closed
EEG: electroencephalography
EMG: electromyography
EO: eyes open
ERD: event-related desynchronization
FDI: first dorsal interosseous
MDT: mechanical detection threshold
MEP: motor evoked potential
MIE: minimally invasive esophagectomy
MVC: maximum voluntary contraction
PPT: pressure pain threshold
QST: quantitative sensory testing
SICI: short-latency intracortical inhibition
SJH: St Joseph’s Healthcare
TMS: transcranial magnetic stimulation
TS: test stimulus
